# A Descriptive Analysis of Dermatology Content and Creators on Social Media in the Philippines

**DOI:** 10.2196/47530

**Published:** 2023-08-21

**Authors:** Kirk Llew Quijote, Arielle Marie Therese Castañeda, Bryan Edgar Guevara, Jennifer Aileen Tangtatco

**Affiliations:** 1 Department of Dermatology Southern Philippines Medical Center Davao City Philippines

**Keywords:** social media, dermatology, dermatologist, creator, content, impact, Philippines, Facebook, Instagram, Twitter, TikTok, YouTube

## Introduction

The use of social media in health information dissemination is an emerging concept, and the quality and reliability of dermatology-related content pose great challenges for creators and end-users, particularly in the Philippines where 80.7% of the population are active social media users [1]. Exposure to and awareness of dermatology health information on social media largely influences the behaviors and practices of populations with access to the internet and social media [2]. Our objective was to describe the content and creators of the most popular Filipino-made dermatology-related image and video posts on Facebook, Instagram, Twitter, TikTok, and YouTube.

## Methods

A web-based search and analysis based on the methods used by Nguyen et al [3] on TikTok were conducted using hashtag terms related to 10 diagnoses (#acne, #alopecia, #cyst, #rash, #eczema, #dermatitis, #tinea, #leprosy, #psoriasis, and #warts), 10 procedures (#botox, #filler, #acnescars, #tattooremoval, #hairremoval, #whitening, #laser, #facelift, #steroidinjection, and #hairtransplant), and 5 general terms (#dermatology, #dermatologist, #boardcertifieddermatologist, #skincare, and #skindisease). The top 40 posts from each of the 25 hashtag queries were sampled from the 5 social media platforms, producing a total of 5000 posts for analysis.

## Results

**Figure 1:**
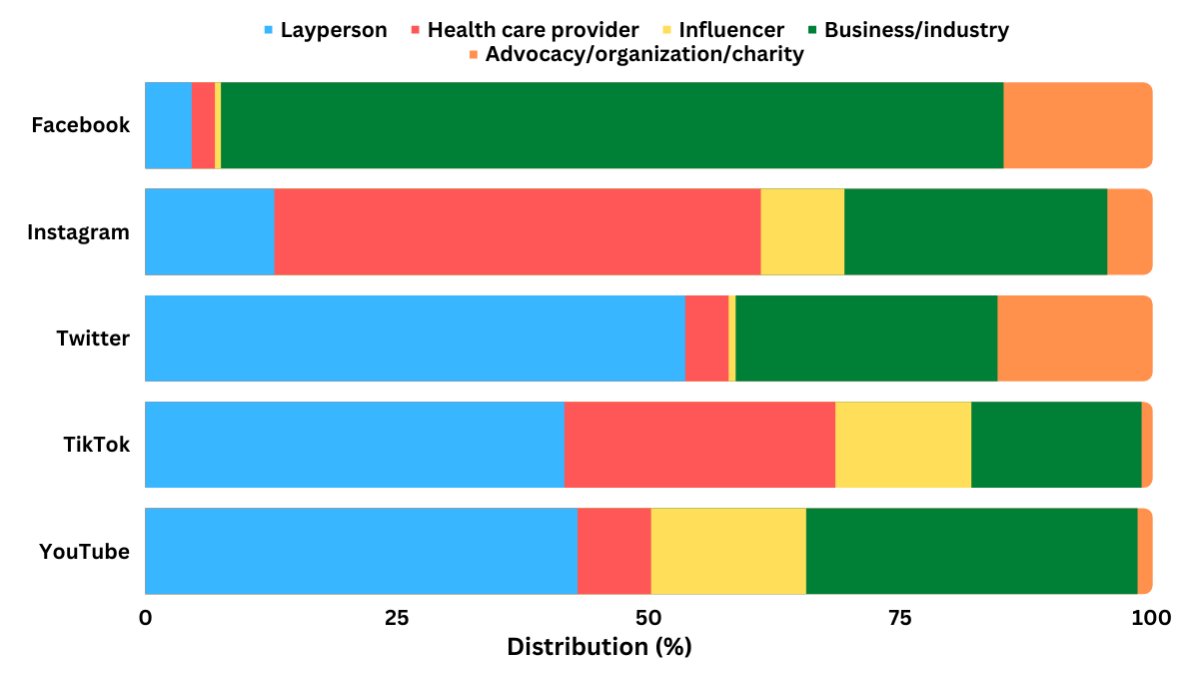
Distribution of content creators (N=2635).

**Figure 2:**
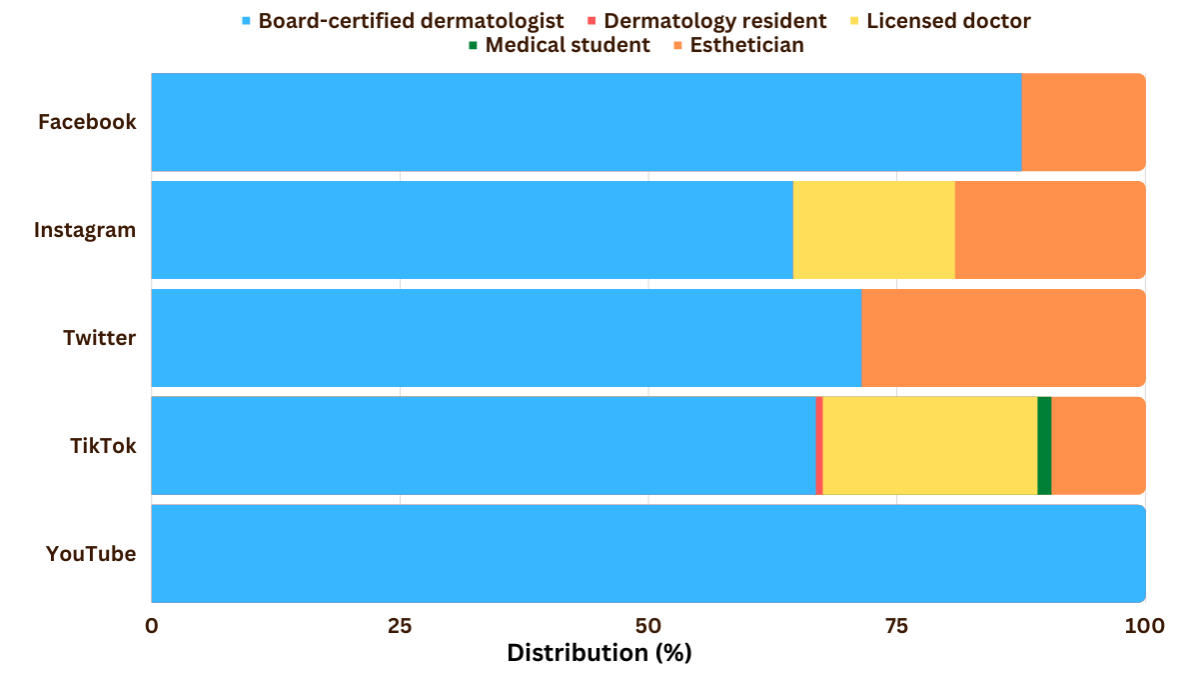
Distribution of health care provider creators (n=465).

Figure 1 shows that more health care providers were identified as creators on Instagram (n=226, 48.1%) and TikTok (n=145, 26.9%) compared to YouTube, Twitter, and Facebook. Specifically, the majority of health care provider creators were board-certified dermatologists (n=154, 64.4% on Instagram; n=99, 66.9% on TikTok; n=25, 71.4% on Twitter; n=7, 87.5% on Facebook; n=36, 100% on YouTube) (Figure 2). This is a substantially higher proportion of board-certified dermatologist creators compared to the findings in previous studies on Instagram where they only comprised 4% of creators [4] but is similar to the findings of studies on TikTok [3] and YouTube [5]. This implies that more Filipino board-certified dermatologists have Instagram and TikTok accounts, making them more visible on these platforms. Laypeople, on the other hand, were the major creators on Twitter, YouTube, and TikTok, implying that dermatology-related data from these platforms may not be reliable or evidence based [2]. Businesses and the pharmaceutical industry comprised the majority of creators on Facebook, implying it is primarily used for business promotions and transactions.

## Discussion

Promotional content comprised the majority of posts across all social media platforms, which contrasted a previous study on TikTok where personal posts by laypeople garnered the highest proportion of creators [3]. Most educational content comprised videos that featured dermatology topics in educational shows, news reports, tutorial videos, and documentaries on skin conditions. This is similar to a study on YouTube [5], where educational videos were found to be more popular among dermatology-related content.

The findings of this study indicate that there is heterogeneity in popular dermatology-related content, creators, and their impacts across social media platforms. Some platforms, such as Instagram, have more health care provider content creators, while others such as Twitter and Facebook, have more laypeople and business/industry creators, respectively. Although board-certified dermatologists make up the majority of health care provider content creators on all platforms, there is still a need to augment their social media presence to further facilitate the provision of evidence-based information. There is also an apparent lack of social media presence from reliable sources on YouTube, Twitter, and Facebook, which necessitates more intervention in these platforms. Promotional content was more common in the majority of search query results in all social media platforms used, followed by educational content, patient experience, and entertainment. Overall, social media truly possesses the power and convenience of access to dermatology health information, and measures to promote and maximize evidence-based content and creators must be implemented, particularly in the Philippines.
